# Silencing of microRNA-708 promotes cell growth and epithelial-to-mesenchymal transition by activating the SPHK2/AKT/β-catenin pathway in glioma

**DOI:** 10.1038/s41419-019-1671-5

**Published:** 2019-06-06

**Authors:** Yan Chen, Xubin Deng, Weiquan Chen, Pengwei Shi, Mei Lian, Hongxiao Wang, Kewan Wang, Dadi Qian, Dong Xiao, Hao Long

**Affiliations:** 1 Department of Neurosurgery, Nanfang Hospital, Southern Medical University, Guangzhou, China; 2 Guangdong Provincial Key Laboratory of Cancer Immunotherapy Research and Guangzhou Key Laboratory of Tumor Immunology Research, Cancer Research Institute, Southern Medical University, Guangzhou, China; 3 Affiliated Cancer Hospital and Institute of Guangzhou Medical University, Guangzhou, China; 4 Department of Emergency, Nanfang Hospital, Southern Medical University, Guangzhou, China

**Keywords:** Cancer, Cell biology

## Abstract

Aberrant microRNA-708 (miR-708) expression is frequently reported in cancer studies; however, its role in glioma has not been examined in detail. We investigated miR-708 function in glioma and revealed that miR-708 expression was significantly down-regulated in glioma tissues and cell lines. Restoration of miR-708 inhibited glioma cell growth and invasion both in vitro and in vivo. The oncogene SPHK2 (sphingosine kinase 2) was identified as a downstream target of miR-708 using luciferase and western blot assays. miR-708 inhibited AKT/β-catenin signaling, which is activated by SPHK2. In addition, we revealed that miR-708 was transcriptionally repressed by EZH2 (enhancer of zeste homolog 2)-induced histone H3 lysine 27 trimethylation and promoter methylation. In summary, our findings revealed that miR-708 is a glioma tumor suppressor and suggest that miR-708 is a potential therapeutic target for glioma patients.

## Introduction

High-grade gliomas, the most common form of brain cancer in adults, are notorious for diffuse invasion and treatment resistance^1,2^. In the past studies, advances have been made in the treatment of glioma with a combination of neurosurgery, radiotherapy, and chemotherapy. However, glioma patients usually have poor prognosis and survival rate^3^, with the 5-year survival rate <5%^4^. It is critical to elucidate the molecular mechanisms involved in glioma progression and new strategies for glioma treatment are urgently required.

MicroRNAs (miRNAs), a class of endogenous non-coding RNAs that are 19–25 nucleotides in length, negatively regulate protein expression by base pairing with 3′-untranslated regions (3′-UTRs)^5,6^. Abnormal expression of miRNAs often contributes to the progression and metastasis of human cancers, including glioma^7,8^. MiRNAs play pivotal role not only in promoting glioma cell growth and invasion but also in leading to chemo- and radio-resistance^9,10^. Several studies have revealed that miR-708 functions as a tumor suppressor, and miR-708 misregulation often contributes to cancer progression^11–13^. However, the role of miR-708 in human glioma is still poorly understood.

Several studies have demonstrated that abnormal AKT signaling activity is associated with tumor progression, including glioma^14^. A recent report documents that AKT promotes glioma cell growth and invasion^15^, and targeting AKT and downstream pathways has been a useful strategy for glioma treatment^16,17^. Elevated AKT activity is correlated with increased β-catenin concentrations. In addition, AKT can lead to β-catenin translocation from the membrane to the nucleus^18^. We previously demonstrated that increased nuclear translocation of β-catenin contributes to accelerated tumor growth and invasion^19,20^. Blocking β-catenin signaling and nuclear β-catenin translocation are essential to inhibit cancer growth and metastasis^21,22^.

Epigenetic silencing through DNA methylation is one of the many mechanisms by which miRNAs suppress cancer in humans. Enhancer of zeste homolog 2 (EZH2), a methyltransferase and the core catalytic element of the polycomb-repressive complex 2 (PRC2), can induce genome-wide histone H3 lysine 27 trimethylation (H3K27me3) and acts as an oncogene by repressing tumor suppressor genes in cancers^23,24^. Recent studies have shown that EZH2 can suppress miRNA expression by inducing H3K27me3 on miRNA promoters^25^.

In this study, we revealed that the expression of miR-708 was down-regulated in glioma cell lines and tissues. Restoration of miR-708 inhibited cell growth and the epithelial-to-mesenchymal transition (EMT) phenotype in glioma, and suppression of the sphingosine kinase 2 (SPHK2)/AKT/β-catenin pathway might be a critical function of miR-708 in glioma. Our study elucidated a link between miR-708 inactivation and the SPHK2/AKT/β-catenin pathway, thereby providing new insight into the potential use of miR-708 in the development of novel therapeutic strategies for treating glioma.

## Results

### miR-708 expression was down-regulated in glioma cell lines and tissues

**Fig. 1:**
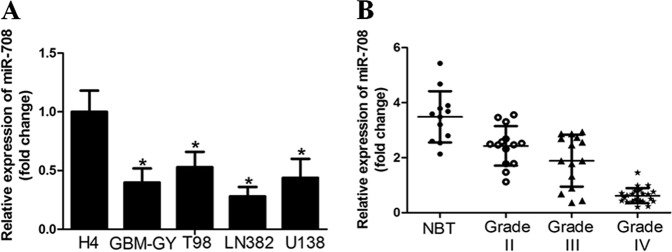
The level of microRNA-708 (miR-708) expression was down-regulated in glioma cell lines and tissues. **a** Reverse transcription-PCR (RT-PCR) assay revealed that miR-708 expression was down-regulated in high-grade glioma cell lines compared to that in low-grade glioma cell line (H4). **b** miR-708 was significantly decreased in glioma samples, as compared to normal brain tissues

First, we examined the expression of miR-708 in glioma cell lines (T98, H4, LN382, U138, and GBM-GY) using quantitative reverse transcription-PCR (qRT-PCR) and observed that miR-708 expression was significantly down-regulated in high-grade glioma cell lines compared to low-grade glioma cell line (H4) (Fig. 1a). The LN382 and GBM-GY cell lines, which had the lowest miR-708 expression, were chosen for subsequent studies. In parallel, we found that miR-708 expression was significantly decreased in glioma tissues compared to normal brain tissues. Interestingly, expression of miR-708 was significantly decreased in high-grade glioma samples (World Health Organization (WHO) tumor grade III and IV), as compared to WHO tumor grade II glioma samples (Fig. 1b).

### Restoration of miR-708 expression inhibited glioma cell growth in vitro and in vivo

The above findings suggested that miR-708 was decreased in high-grade glioma and may be a tumor-suppressive microRNA.

Thus, we determined whether the restoration of miR-708 expression inhibited glioma cell growth and invasion. We established LN382 and GBM-GY cells stably expressing miR-708, and the efficiency of transduction was validated (Supplementary Fig. 1A).

**Fig. 2:**
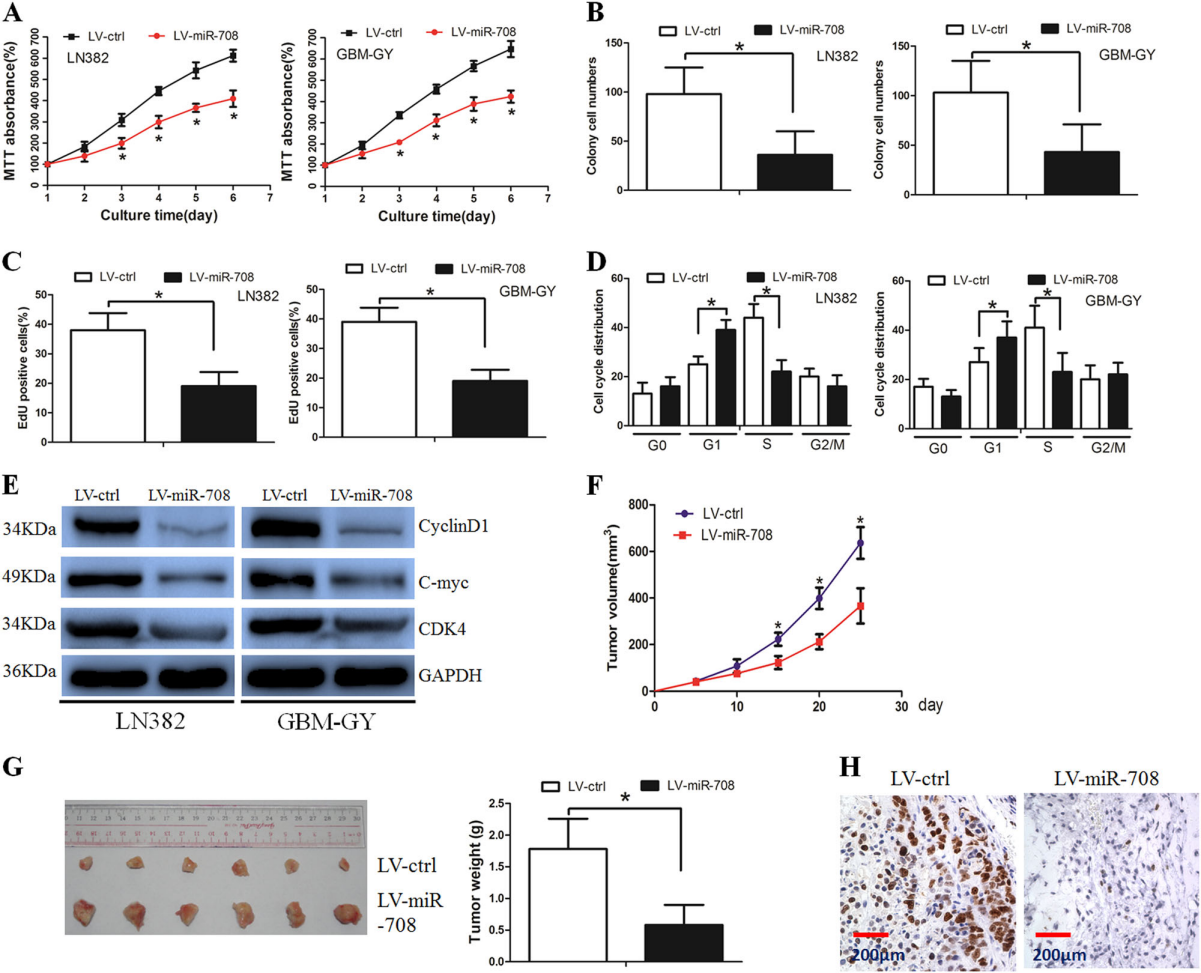
Overexpression of microRNA-708 (miR-708) decreased glioma cell growth both in vitro and in vivo. **a** Cell viability was decreased in the LV-miR-708 group when compared with that in the LV-ctrl group. **b** Restoration of miR-708 decreased LN382 and GBM-GY cell proliferation, as determined by colony formation assay. **c** 5-Ethynyl-2′-deoxyuridine (EdU) assay revealed that the number of LV-miR-708 cells stained with EdU was less than that of LV-ctrl cells stained with EdU. **d** Cell cycle was arrested in the G1 phase when miR-708 was overexpressed in glioma cells. **e** Western blot assay present the altered G1/S-phase checkpoint protein expression in the LV-miR-708 group. **f** Glioma cells in the LV-miR-708 group grew slower than cells in the LV-ctrl group. **g** Xenograft tumors from the LV-miR-708 group were smaller than that from the LV-ctrl group. **h** Immunohistochemical (IHC) assay revealed that the Ki-67 staining index was decreased in the LV-miR-708 group when compared with that in the LV-ctrl group

The 3-(4,5-dimethylthiazol-2-yl)-2,5-diphenyltetrazolium bromide (MTT) assay revealed that miR-708 significantly decreased cell viability when compared with the LV-ctrl group (Fig. 2a). The colony formation assay showed that miR-708 overexpression inhibited cell proliferation (Fig. 2b, Supplementary Fig. 1B). In parallel, the number of cells incorporating 5-ethynyl-2′-deoxyuridine (EdU) was significantly decreased in the LV-miR-708 group compared with the LV-ctrl group (Fig. 2c, Supplementary Fig. 1C). Next, we performed flow cytometry to examine whether miR-708 inhibited glioma cell proliferation by altering cell cycle progression and observed a higher proportion of LV-miR-708 cells in the G1 phase and a lower proportion in the S phase compared to the control (Fig. 2d, Supplementary Fig. 1D). In addition, western blotting revealed that the expression of G1/S-phase checkpoint proteins (e.g., c-myc, cyclin D1, and CDK4) were significantly decreased in LV-miR-708 cells (Fig. 2e). Overall, these data suggest that miR-708 inhibited glioma cell growth through affecting cell cycle progression.

A tumorigenesis study was performed to confirm that miR-708 inhibited tumor growth in vivo. LN382 cells were used for the in vivo study. We revealed that miR-708-transduced cell lines present markedly slower growth after implantation (Fig. 2f). After the mice were scarified, xenograft tumors were removed from the mice and weighted. The mean weight of xenograft tumors from miR-708-transduced cells was also significantly less than that from the control cells (Fig. 2g). The proliferation index of the xenograft tumors was then examined by performing Ki-67 expression staining analysis (Fig. 2h). Taken together, these data suggested that miR-708 inhibited glioma cell tumorigenesis in vivo.

### miR-708 inhibited glioma cell invasion by reversing the EMT phenotype

**Fig. 3:**
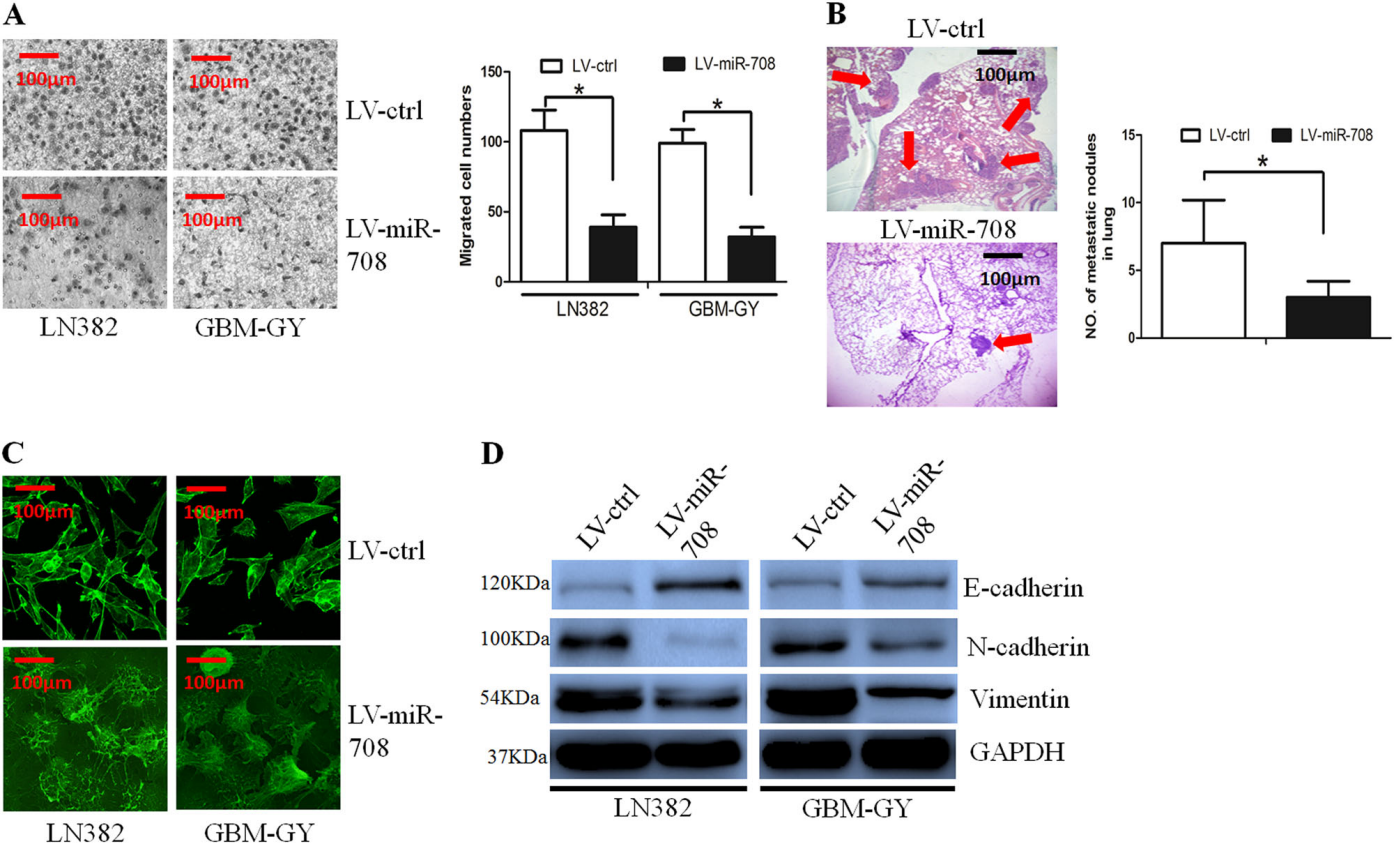
Overexpression of microRNA-708 (miR-708) decreased glioma cell invasion through reversing the epithelial-to-mesenchymal transition (EMT) phenotype. **a** The Boyden assay revealed that glioma cell invasion ability was decreased in the LV-miR-708 group. **b** The in vivo assay revealed that miR-708 decreased the number and size of metastatic pulmonary nodules. **c** The formation of stress fiber in glioma cells was decreased in the LV-miR-708 group. **d** The epithelial marker E-cadherin was increased, whereas expression of the mesenchymal markers N-cadherin and vimentin were decreased in the LV-miR-708 group, as determined by western blot assay

We wanted to determine whether miR-708 affected glioma cell invasion using the Boyden assay, which revealed that miR-708 overexpression inhibited glioma cell invasion (Fig. 3a). Furthermore, an in vivo metastasis assay was performed to analyze whether miR-708 inhibited glioma cell invasion in vivo. The results revealed that miR-708 expression resulted in a significant decrease in the number and size of pulmonary metastatic nodules (Fig. 3b).

Cytoskeletal reorganization, exemplified by the formation of stress fiber bundling arrays, plays significant role in cancer cells invasion^26^. The phalloidin staining assay revealed that miR-708 decreased formation of stress fiber in glioma cells (Fig. 3c).

EMT is implicated in cancer progression and metastasis; we then examined whether miR-708 affected the EMT phenotype. Using western blotting, we compared the expression of epithelial and mesenchymal markers between the LV-miR-708 and LV-ctrl cells. We observed that when miR-708 expression was restored in glioma cells, expression of the epithelial marker E-cadherin was increased, whereas expression of the mesenchymal markers N-cadherin and vVimentin were decreased (Fig. 3d).

Overall, these results suggest that restoration of miR-708 can reverse the EMT phenotype in glioma cells.

### SPHK2 was a direct target of miR-708

**Fig. 4:**
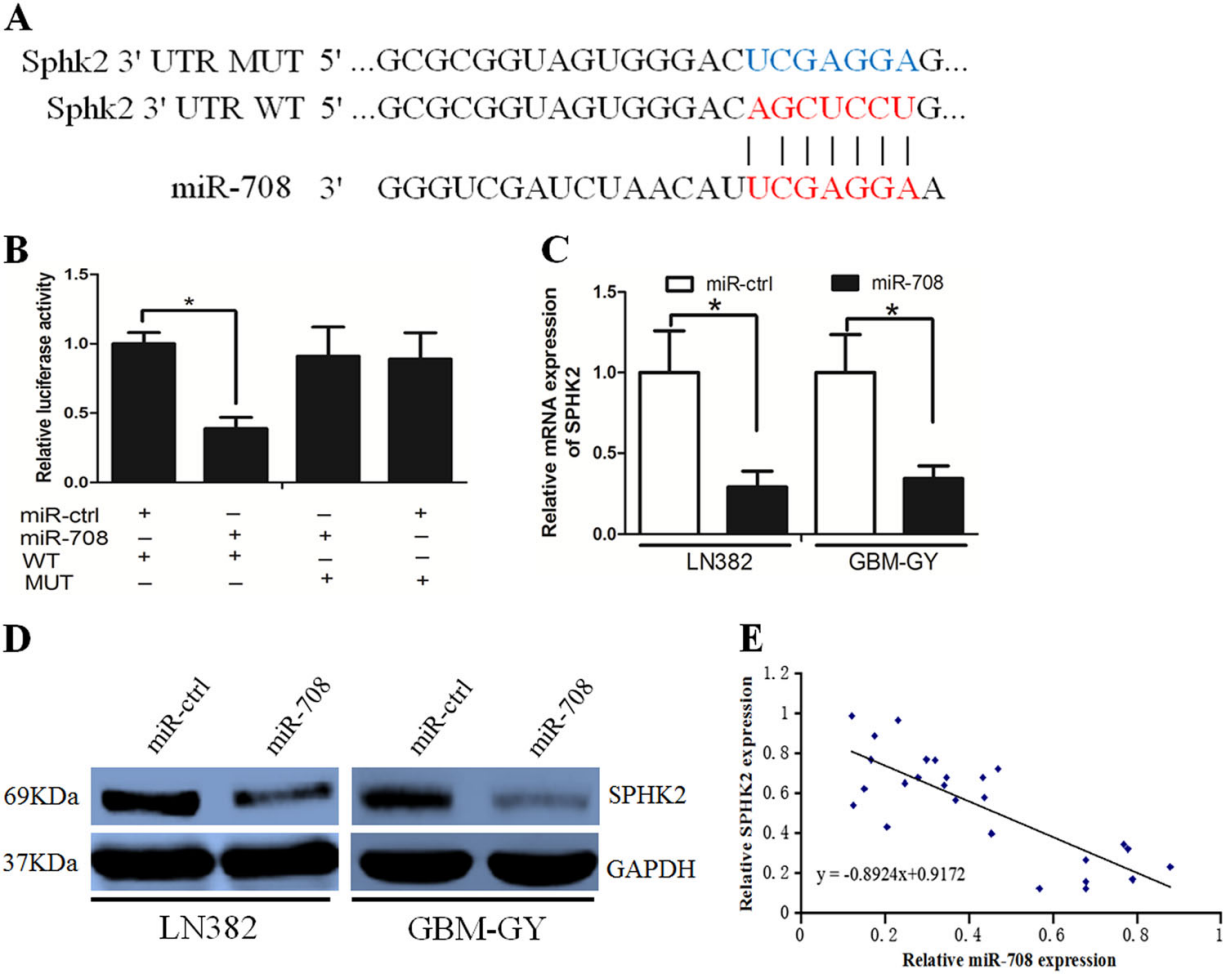
MicroRNA-708 (miR-708) directly targeted sphingosine kinase 2 (SPHK2) in glioma cells. **a** Binding sites of miR-708 at SPHK2 3′-untranslated region (3′-UTR), SPHK2 wild-type (WT), and mutant (MT) 3′-UTR are indicated. **b** The luciferase activity in SPHK2 wild-type construct, instead of luciferase activity in mutant construct, was decreased when miR-708 was presented. **c** miR-708 decreased SPHK2 messenger RNA (mRNA) expression level in LN382 and GBM-GY cells. **d** miR-708 decreased SPHK2 protein levels in LN382 and GBM-GY cells. **e** The reversed association between miR-708 and SPHK2 expression was indicated in glioma tissues

Using bioinformatic analyses (TargetScan and miRanda), SPHK2 was predicted to be a possible miR-708 target. A luciferase reporter assay was performed to determine whether miR-708 directly targeted the SPHK2 3′-UTR. We subcloned the SPHK2 3′-UTR (including the predicted miR-708 recognition site (wild-type (WT)) or a mutated sequence (mutant (MUT) type)) from messenger RNA (mRNA) into luciferase reporter plasmids (Fig. 4a). A significant decrease in luciferase activity was detected in LN382 cells transfected with the WT miR-708 vector compared with cells transfected with the MUT vector (Fig. 4b). Subsequently, we assessed whether miR-708 inhibited SPHK2 expression using RT-PCR and western blotting and revealed that miR-708 decreased both mRNA and protein SPHK2 levels in LN382 and GBM-GY cells (Fig. 4c, d). However, when the biding sites of miR-708 on SPHK2 were MUT, the suppressive effects of miR-708 on SPHK2 mRNA and protein were abolished (Supplementary Fig. 1E, F). Interestingly, we also observed a negative association between miR-708 and SPHK2 expression in glioma tissues (Fig. 4e).

**Fig. 5:**
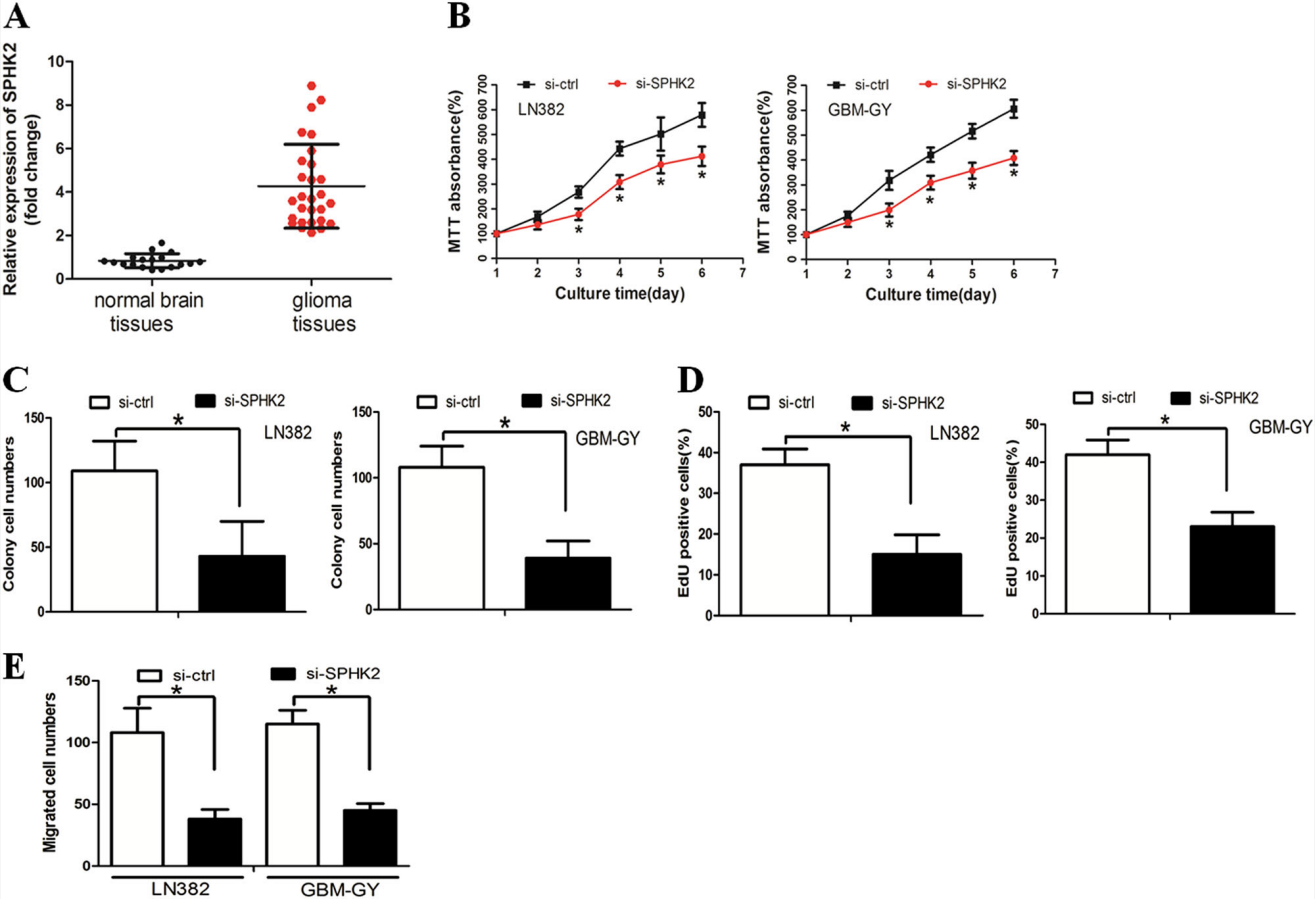
Sphingosine kinase 2 (SPHK2) was a glioma oncogene. **a** SPHK2 expression was elevated in glioma tissues compared with the normal brain tissues. **b** 3-(4,5-Dimethylthiazol-2-yl)-2,5-diphenyltetrazolium bromide (MTT) assays revealed that SPHK2 down-regulation inhibited glioma cell growth. **c** The colony-forming ability of glioma cells was decreased after restoration of microRNA-708 (miR-708) expression. **d** The number of 5-ethynyl-2′-deoxyuridine (EdU)-positive cells was decreased in the LV-miR-708 group compared with the LV-ctrl group. **f** Boyden assay demonstrated that miR-708 inhibited glioma cell invasion

We investigated the underlying role of SPHK2 in glioma. RT-PCR revealed that SPHK2 expression was elevated in glioma tissues compared with normal brain tissues (Fig. 5a). We explored the functional impact of SPHK2 in glioma cells by knocking down SPHK2 expression with small interfering RNA (siRNA) in LN382 and GBM-GY cells. We observed that SPHK2 down-regulation inhibited glioma cell growth and invasion (Fig. 5b–e). Taken together, these data suggest that SPHK2 functions as an oncogene in glioma cells.

Furthermore, we asked whether SPHK2 overexpression reversed the suppressive effects of miR-708 exhibited on cell growth and invasion. We simultaneously co-transfected glioma cells with a miR-708 mimic and a pcDNA3.1-SPHK2 vector that encoded full-length SPHK2 except for its 3′-UTR. The results showed that cells expressing the pcDNA3.1-SPHK2 vector reversed the miR-708-mediated decrease of SPHK2 expression (Supplementary Fig. 2A). In addition, SPHK2 overexpression counteracted the effect of miR-708 on glioma cell growth and invasion (Supplementary Fig. 2B–G). However, treatment with pcDNA3.1 vector did not rescue the effects of miR-708 on SPHK2 expression (Supplementary Fig. 2A). And pcDNA3.1 vector treatment did not counteract the effects of miR-708 on glioma cell growth and invasion (Supplementary Fig. 2B–G).

### AKT/β-catenin signaling mediated the effects of miR-708 on glioma cells

SPHK2 activates the AKT signaling pathway by increasing AKT phosphorylation^27^, which in turn activates the WNT/ β-catenin pathway by phosphorylating and inhibiting glycogen synthase kinase 3-β (GSK3-β) activity. Phosphorylation of β-catenin by AKT increases its transcriptional activity and promotes tumor cell invasion and growth^18^. We explored whether the AKT/ β-catenin axis mediated the effects of miR-708.

**Fig. 6:**
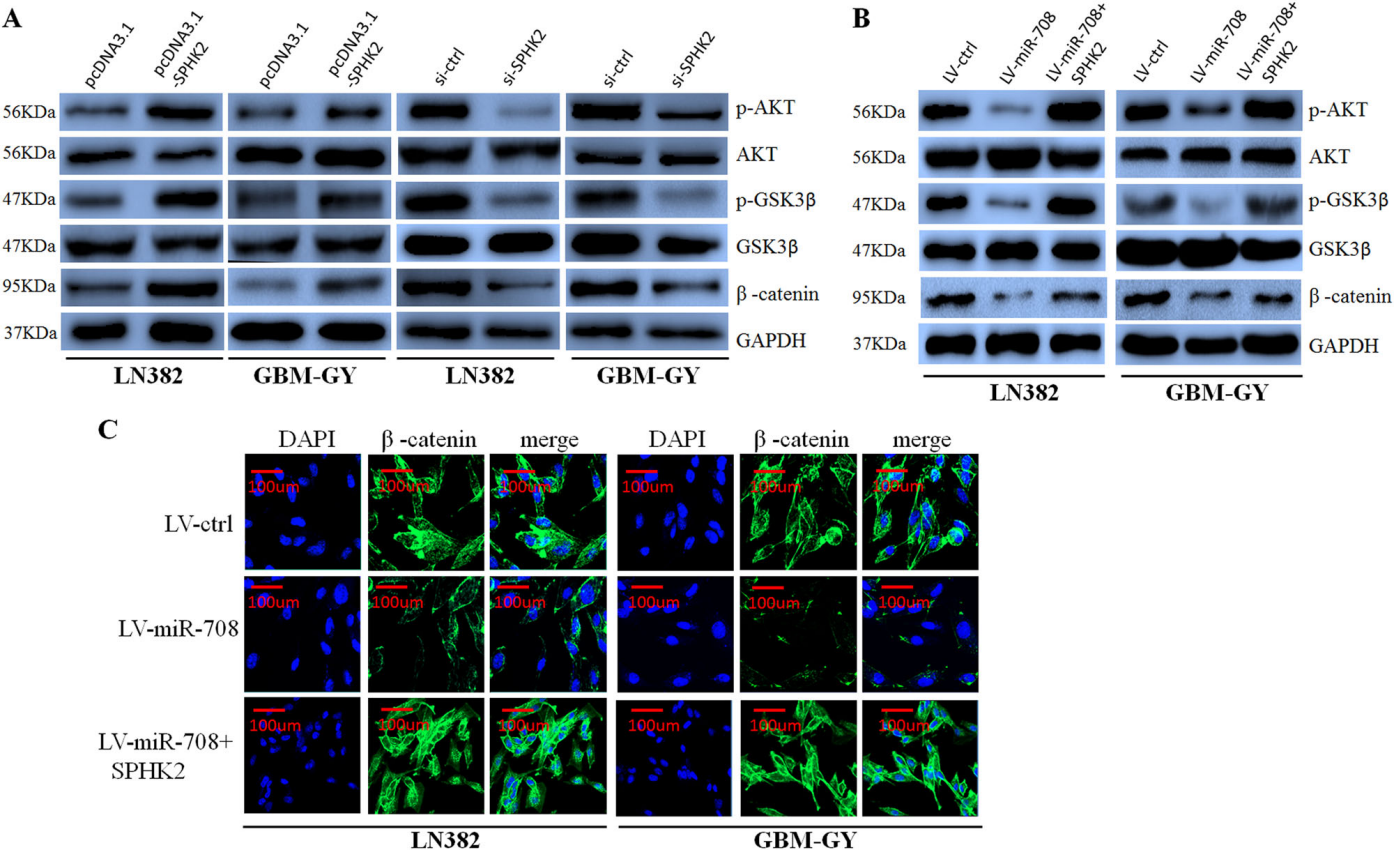
MicroRNA-708 (miR-708) inhibits the AKT/β-catenin pathway. **a** Overexpression of sphingosine kinase 2 (SPHK2) elevated phosphorylated (p)-AKT, p-glycogen synthase kinase 3-β (GSK3-β), and β-catenin expression, whereas anti-SPHK2 has the opposite effect. **b** Restoration of SPHK2 expression resulted in the recovery of p-AKT, p-GSK3-β, and β-catenin expression that had been decreased by miR-708 transfection. **c** Immunofluorescence assays revealed that SPHK2 enhanced nuclear β-catenin expression that had been repressed by miR-708

First, we found that SPHK2 overexpression increased phosphorylated Akt, phosphorylated GSK3-β and β-catenin levels, whereas SPHK2 inhibition decreased phosphorylated Akt, phosphorylated GSK3-β, and β-catenin levels in glioma cells (Fig. 6a). However, western blotting showed that miR-708 expression inhibited the expression of SPHK2, phosphorylated Akt, phosphorylated GSK3-β, and β-catenin, which was counteracted by SPHK2 overexpression in LN382 and GBM-GY cells (Fig. 6b). Interestingly, immunofluorescence also revealed that miR-708 prevented the nuclear accumulation of β-catenin, which was restored by SPHK2 overexpression (Fig. 6c).

To further determine whether β-catenin mediated the effects of miR-708 on glioma cells, we decreased β-catenin levels in LN382 and GBM-GY cells using siRNA (Supplementary Fig. 3A). We revealed that β-catenin knockdown mimicked the effect of miR-708 on glioma cells (Supplementary Fig. 3B–G).

Together, these data suggest that the AKT/β-catenin axis might mediate the effect of miR-708 on glioma cell growth and invasion.

### EZH2 repressed miR-708 expression in glioma cells

**Fig. 7:**
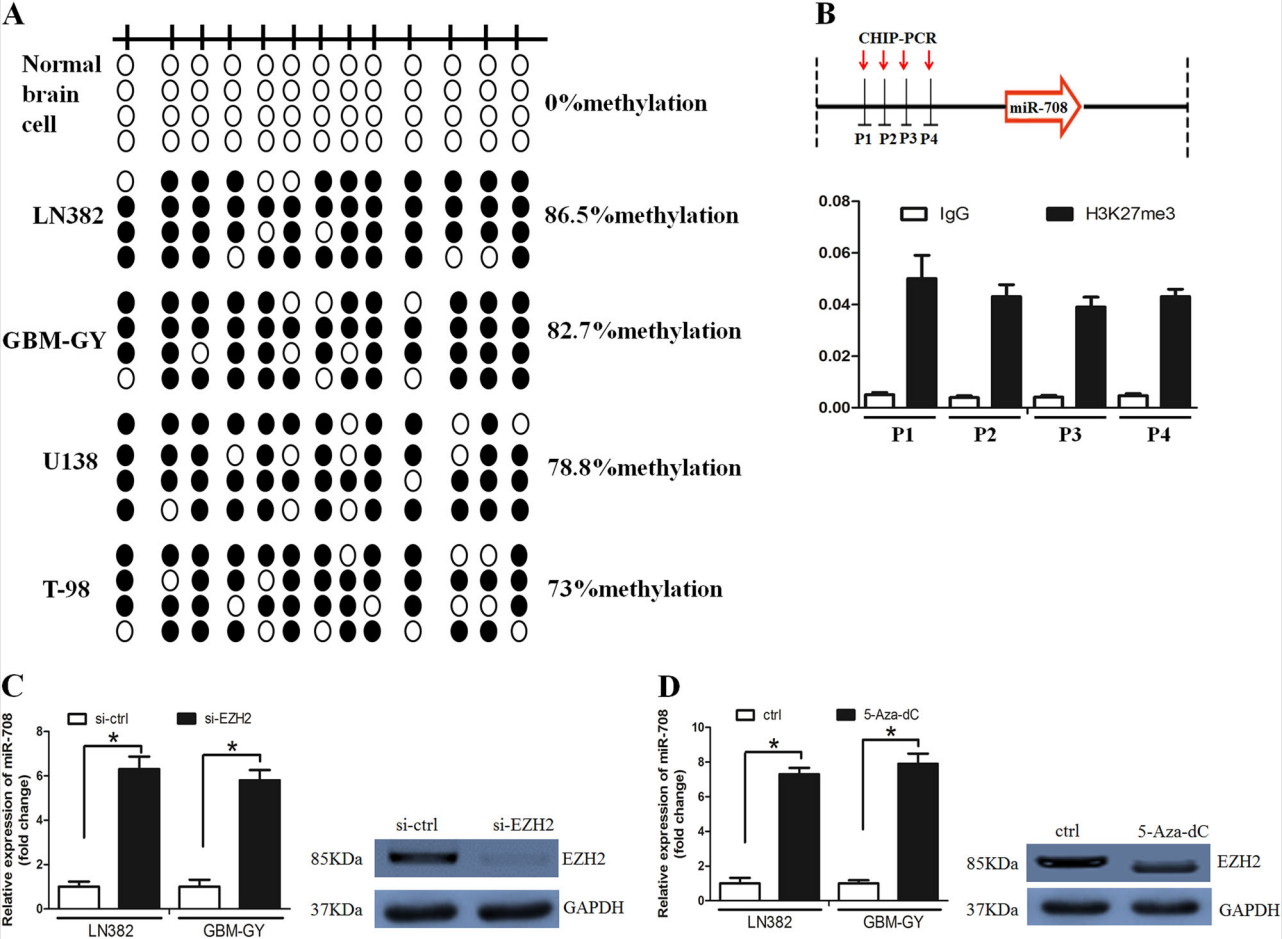
Enhancer of zeste homolog 2 (EZH2) inhibited microRNA-708 (miR-708) expression. **a** The methylation status of the miR-708 promoter was detected in normal brain cell line and glioma cell lines. **b** Histone H3 lysine 27 trimethylation (H3K27me3) was enriched around the miR-708 promoter region. **c** EZH2 down-regulation increased miR-708 expression in LN382 and GBM-GY cell lines. **d** Treated with deazaneplanocin A (DZNep) elevated miR-708 expression in LN382 and GBM-GY cell lines

Promoter methylation often contributes to microRNA down-regulation. We thus examined the promoter status of miR-708 with using bisulfite sequencing PCR (BSP) assay. It was revealed that miR-708 methylation was higher in glioma cell lines compared with NBC (Fig. 7a).

Subsequently, chromatin immunoprecipitation (ChIP) assay was used to examine H3K27me3 levels around the miR-708 promoter region. It was found that H3K27me3 was highly enriched around the miR-708 promoter (Fig. 7b). Interestingly, EZH2 down-regulation or treatment with deazaneplanocin A (DZNep) increased miR-708 levels (Fig. 7c, d). Taken together, our findings suggest that miR-708 was epigenetically silenced by DNA methylation and EZH2-mediated histone methylation in glioma.

In addition, we found that EZH2 led to activation of SPHK2 and AKT and contributed to cell growth, invasion, and EMT phenotype. Restoration of miR-708 could counteract EZH2 overexpression effect on glioma cell growth and invasion (Supplementary Fig. 4A–D).

### miR-708 was associated with poor prognosis in glioma patients and was negatively associated with EZH2 expression

**Fig. 8:**
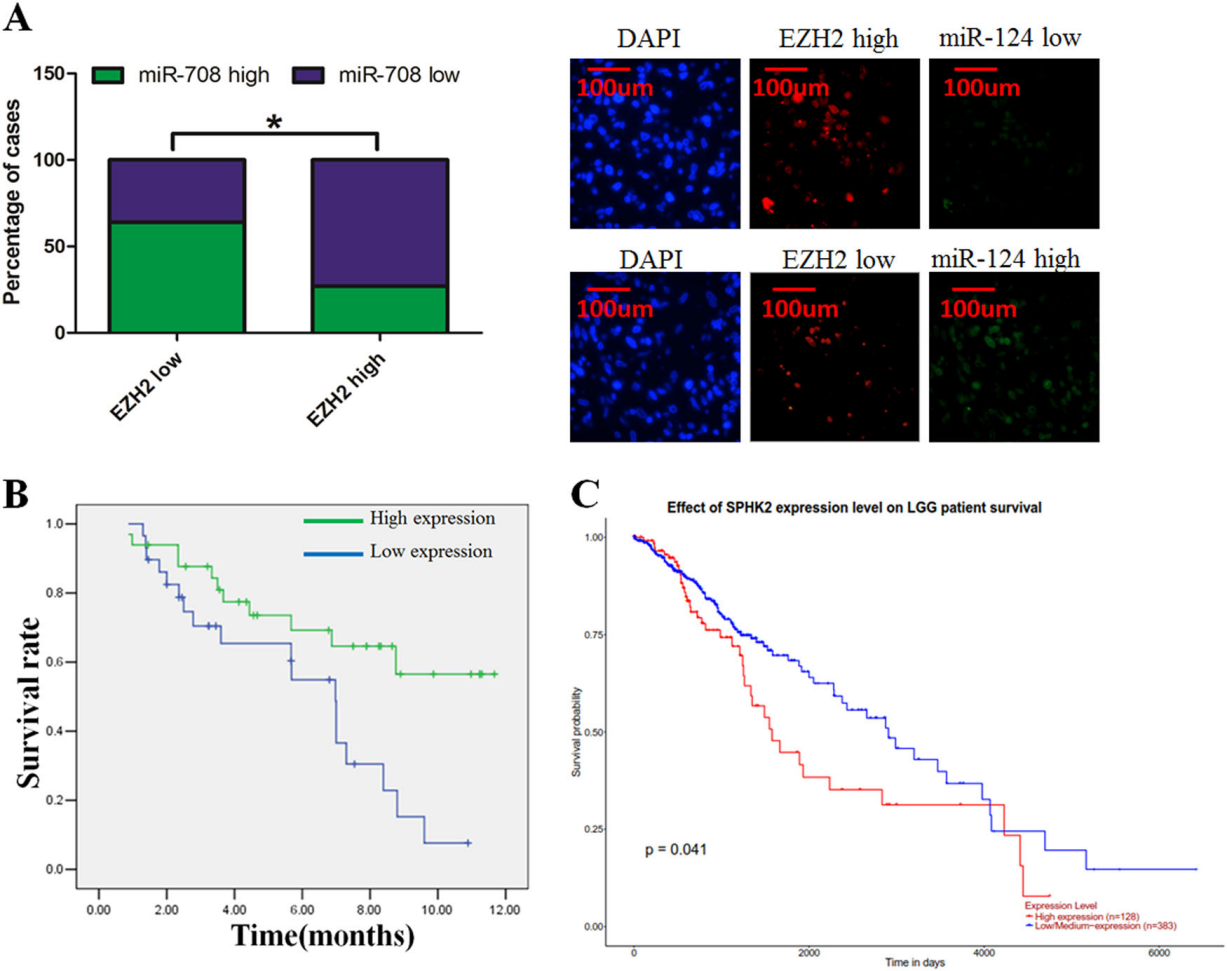
microRNA-708 (miR-708 predicted poor glioma patient prognosis and was negatively associated with enhancer of zeste homolog 2 (EZH2) expression. **a** We observed a negative relationship between miR-708 and EZH2 expression (left panel). In situ hybridization results showing miR-708 and EZH2 expression levels in glioma tissues (right panel). **b** Glioma patients with low miR-708 levels display worse overall survival rates than those with high levels of miR-708 in high-grade glioma patients. **c** The TCGA database revealed that low miR-708 levels display worse overall survival rates than those with high levels of miR-708 in low-grade glioma patients

We analyzed the relationship between clinicopathological characteristics and miR-708 expression in a large cohort of 99 glioma patients. There was no significant association between the miR-708 expression and gender, age, or histological type. However, we revealed that miR-708 expression levels were associated with clinical stage (I–II compared to III–IV; *P* = 0.017) (Table S1). We detected the expression of miR-708 and EZH2 in glioma tissues using in situ hybridization and observed a negative relationship between miR-708 and EZH2 expression, which was quantified using Spearman’s correlation analysis (Fig. 8a). Interestingly, high-grade glioma patients (WHO tumor grade III and IV) with low levels of miR-708 exhibited poorer survival than those with high miR-708 levels (Fig. 8b). We also analyze the transcript reads of SPHK2 from the The Cancer Genome Atlas (TCGA) database. However, we revealed that SPHK2 was significantly associated with overall survival rate in lower grade glioma patients, instead of that in higher grade glioma patients (Fig. 8c).

## Discussion

Accumulating evidence has documented that miRNAs are involved in cancer development and progression, including glioma^28,29^. Previous studies presented that miR-708 was usually down-regulated in various types of cancer. For example, miR-708 regulates chemoresistance in human non-small-cell lung cancer through the EMT^30^. Similarly, abnormal expression of miR-708 contributes to the incidence of breast cancer^31^, bladder cancer^32^, and larynx carcinoma^33^. However, the biological function and underlying mechanisms of miR-708 function in glioma are still poorly understood. Our findings present that the expression level of miR-708 was down-regulated in glioma tissues and cell lines. Subsequently, we demonstrated that high-grade glioma usually had low-level expression of miR-708. In addition, we analyzed the relation between miR-708 expression and overall survival rate in glioma patients (Fig. 8b). In low-grade glioma (WHO I–II grade), we found that the expression level of miR-708 was not significantly associated with survival rate. (Because we did not find significant association between miR-708 expression level and survival rate in these patients, we did not present the data.) However, in high-grade gliomas (WHO III–IV grade), patients with high expression of miR-708 exhibited better survival than those with low miR-708 levels. We propose that miR-708 may be a suitable prognosis within high-grade gliomas instead of low-grade gliomas. Notably, we analyze the transcript reads of SPHK2 from the TCGA database. However, we revealed that SPHK2 was significantly associated with overall survival rate in lower grade glioma patients, instead of that in higher grade glioma patients. This result conflicted with our data. We speculated that the conflict between our data and TCGA database was due that the patients were from different regions and they have different expression levels of SPHK2. Further investigation is needed to clarify the underlying mechanism. IDH1 mutations in glioma have different clinical outcomes^34^. However, we did not examine IDH1 mutations in these patient samples. This is the limitation of our research. Further investigation is urgently required to clarify the association between miR-708 and IDH1 mutations in glioma.

In further study, we expressed miR-708 in glioma cells and showed that restoring miR-708 inhibited glioma cell growth both in vitro and in vivo by inducing cell cycle arrest at the G1/S transition. Furthermore, the restoration of miR-708 inhibited glioma cell invasion both in vitro and in vivo. The EMT phenotype is responsible for cancer cell metastasis, including in glioma^35^. We observed that miR-708 overexpression reversed the EMT phenotype in glioma cells. Zhang et al.^36^ found that miR-708 restricted cell migration and invasion in lung adenocarcinoma, and Gao et al.^33^ reported that miR-708 inhibited larynx carcinoma cell metastasis. In ovarian cancer, miR-708 suppressed metastasis by targeting the Rap1B protein^37^, and in breast cancer, overexpression of miR-708 modulated cell metastasis by regulating the EMT phenotype^31^. Our findings, which agree with other published reports, suggest that miR-708 functions as a tumor suppressor in glioma.

Subsequently, we identified that SPHK2 was a direct target of miR-708. miR-708 negatively regulated the expression of SPHK2, as determined by luciferase reporter and western blot assays. SPHK2 is an architectural transcription factor that plays a crucial role in the development and progression of various malignant cancers. Abnormal SPHK2 expression usually promotes cancer cell proliferation and invasion^38^, and SPHK2 protein expression is frequently elevated in glioma tissues and correlates with poor patient survival^39,40^. We revealed that SPHK2 down-regulation inhibited glioma cell growth and invasion. Because SPHK2 down-regulation mimicked the effect of miR-708 on glioma cells, we hypothesized that miR-708 directly targets SPHK2. To examine this possibility, we re-introduced SPHK2 into the LV-miR-708 cells and observed that SPHK2 overexpression reversed the tumor-suppressing effects of miR-708 on glioma cells. Interestingly, we also found that there was a negative correlation between miR-708 and SPHK2 expression in glioma tissues. These observations suggest that miR-708 exerts its function in glioma by mainly targeting SPHK2.

SPHK2 activates the AKT signaling pathway^41^, and consistent with previous findings, we observed that SPHK2 overexpression increased p-AKT levels, whereas SPHK2 knockdown had the opposite effect. AKT might phosphorylate β-catenin by inhibiting GSK3β, which ubiquitinates and degrades β-catenin. AKT-mediated phosphorylation of β-catenin causes it to disassociate from cell–cell contacts and accumulate in both the cytosol and the nucleus, and β-catenin phosphorylation by AKT increases its transcriptional activity and promotes tumor cell invasion^18^. The activation of β-catenin has been shown to contribute to cell invasion and growth in glioma^42^, whereas inhibition of β-catenin signaling reverses the EMT phenotype in glioma^43,44^. Our study found that miR-708 inhibited the activation of the AKT/β-catenin signaling pathway by negatively regulating SPHK2. In addition, our findings revealed that β-catenin knockdown decreased glioma cell growth and invasion. Therefore, we propose that the SPHK2/AKT/β-catenin axis mediates the effect of miR-708 on glioma cell growth and invasion.

A previous study revealed that miR-708 was regulated by the PcG repressor complex^45^, an observation supported by a subsequent study^46^. PRC1 and PRC2 are two distinct PcG complexes. PRC2, which contains the SUZ12 subunit and the catalytic EZH2 subunit, contributes to gene repression by regulating H3K27me3^47^. EZH2, which usually overexpressed in serious types of cancer, contributes to tumorigenesis through regulating microRNA expression. For instance, EZH2 bides to the promoter regions of miR-1246, miR-302a, and miR-4448 and represses their expression^48^. Similarly, it was observed that EZH2 repressed a panel of microRNAs and promoted cancer cell growth and invasiveness^49^. A recent study revealed that PRC2-induced H3K27me3 contributed to the down-regulation of miR-708 in metastatic cancer cells^50^. We asked whether miR-708 down-regulation in glioma was due to promoter methylation. It was found that H3K27me3 mediated EZH2’s suppressive effect on miR-708. Inhibition of EZH2 or treatment with DZNep can elevate miR-708 expression in LN382 and GBM-GY cells.

In summary, our data provide the first evidence that the EZH2/miR-708/SPHK2/AKT/β-catenin axis controls growth and invasion in glioma cells. Despite the limitation in our study, targeting miR-708 in glioma may be a promising direction to purse.

## Materials and methods

### Glioma cell culture and primary glioma cell establishment

Human glioma cell lines (H4, T98, LN382, and U138) were purchased from the Institute of Biochemistry and Cell Biology of the Chinese Academy of Sciences (Shanghai, China). The cells were cultured with Dulbecco’s modified Eagle’s medium (DMEM) supplemented with 10% fetal bovine serum (FBS) under 37 °C atmosphere containing 5% CO_2_.

To establish primary glioma cell line GBM-GY, glioma tissues were minced in DMEM/Ham’s F12 cell culture media (contains penicillin–streptomycin, 10% FBS, and 2 mM l-glutamine), followed by filtering with a cell strainer. The cell suspension was then washed with phosphate-buffered saline and cultured in 24-well plates coated with collagen, followed by culturing in T25 cell culture flasks and expanded for subsequent analyses. The GBM-GY cells were then cultured with DMEM supplemented with 10% FBS under 37 °C atmosphere containing 5% CO_2_.

### Patient sample collection and ethics statement

A total of 99 patients were enrolled in this study. The information on clinical characteristics of patients used in this study was summarized in Supplementary Table S1. All the clinical specimens were obtained with informed consent and approved by the Ethical Committee of the Guangzhou Medical University and Southern Medical University. Informed consent was obtained from all patients involved in this study. Animal experiments were reviewed and approved by the Institutional Animal Care and Use Committee of the Guangzhou Medical University and Southern Medical University. All the experiments were performed in accordance with the approved guidelines of the Institutional Research Ethics Committee of the Guangzhou Medical University and Southern Medical University.

### Lentivirus production and infection

We obtained pLV-has-miR-708 and pLV-miRNA (negative control) plasmids from Biosettia Inc. (San Diego, CA, USA). Retroviral production and infection were performed as previously described^8^. The packaged lentiviruses were named as LV-miR-708 and LV-ctrl accordingly.

### Cell transfection

miR-708 mimics and the negative control miR-ctrl were synthesized by GenePharma (Shanghai, China). Cell transfection was carried out as previously described^51^.

### Dual luciferase activity assay

Full-length SPHK2 cDNA without the 3′-UTR was subcloned into the eukaryotic expression vector pcDNA3.1 (Invitrogen). We amplified the 3′-UTR from SPHK2 and cloned it downstream of the firefly luciferase gene in the pGL3 vector (Promega). We named the vector WT SPHK2 3′-UTR. Site-directed mutagenesis of the miR-708 binding sites in the SPHK2 3′-UTR was performed using the GeneTailor Site-Directed Mutagenesis System (Invitrogen). We named the vector MUT SPHK2 3′-UTR. Cells were co-transfected with the WT or MUT SPHK2 3′-UTR vector and the miR-708 mimic or inhibitor. The luciferase assay was performed with the Dual Luciferase Reporter Assay System (Promega) 48 h after transfection.

### qRT-PCR and western blot assays

We extracted total RNA by using TRIzol reagent (Invitrogen). The primers used were listed in Supplementary Table S2. MiR-708 expression was measured by using the TaqMan MicroRNA Assay. U6 small nuclear RNA was used as a normalization control. SPHK2 and glyceraldehyde 3-phosphate dehydrogenase (GAPDH) expressions were measured by using the SYBR Green System. The relative expression of SPHK2 was calculated and normalized to GAPDH using the 2^−ΔΔCt^ method.

For the western blot assay, proteins were extracted from the cells, resolved using sodium dodecyl sulfate-polyacrylamide gel electrophoresis and transferred to polyvinylidene fluoride membranes. Subsequently, the membranes were blocked in 5% non-fat skim milk/TBST (Tris-buffered saline, 0.1% Tween-20) and then incubated with primary antibodies at 4 °C overnight. Finally, the membranes were incubated with the appropriate secondary antibodies, and the levels of total protein were detected using enhanced chemiluminescence reagents. The primary antibodies cyclin D1 (Lot No. ab134175), c-myc (Lot No. ab32072), CDK4 (Lot No. ab108357), E-cadherin (Lot No. ab1416), N-cadherin (Lot No. ab202030), vimentin (Lot No. ab92547), SPHK2 (Lot No. ab37977), p-AKT (Lot No. ab131443), AKT (Lot No. ab38449), p-GSK3-β (Lot No. ab107166), GSK3-β (Lot No. ab32391), β-catenin (Lot No. ab16051), and GAPDH (Lot No. ab181602) were purchased from Abcam. The concentration used in the study was 1:500.

### Fluorescent in situ hybridization

In situ detection of miR-708 was performed as previously described^52^. Briefly, tissue sections were baked, deparaffinized, and fixed in 4% paraformaldehyde. Then, the tissues were prehybridized in hybridization buffer, followed by treatment with double digoxigenin-labeled locked nucleic acid-modified probe corresponding to mature miR-708. Subsequently, the tissues were incubated in anti-digoxigenin-horse radish peroxidase antibody for 1 h at room temperature. Finally, images were acquired with a confocal microscope.

### MTT, EdU, and Boyden assays

The MTT assay was performed based on previously described methods^53^. Briefly, cells were seeded on 96-well plate and were allowed to grow for 24 h. Then, the media were aspirated and MTT solution was added into each well. After incubation for 30 min, 150 µL of dimethyl sulfoxide was added into each well. Finally, the absorbance was read at optical density equal to 590 nm.

The EdU incorporation assay was carried out using the Cell-Light TM EdU Imaging Detecting Kit according to the manufacturer’s instructions (RiboBio, Guangzhou, China).

The Boyden assay was carried out as previously described^51^. Briefly, the cells were seeded into the upper chambers (Millipore) that were coated with 150 µg Matrigel (BD Biosciences, Boston, MA, USA). Under the upper chambers were lower chambers, which were filled with 500 µL DMEM supplemented with 10% FBS. After incubation for 12 h, the cells adhering to the lower surface were fixed with methanol, stained with Giemsa solution, and counted.

### In vivo tumor growth and invasion assay

All procedures involving animals were approved by the Institutional Committee on Animal Care at Guangzhou Medical University. Cells were mixed with Growth Factor Reduced Phenol Red-Free Matrigel and injected subcutaneously into both flanks of nude mice. Four weeks after implantation, the xenografts were removed from the mice and weighed. The tumor volume was calculated using the following formula: 4*π*/3 × (width/2)^2^ × (length/2). The invasion assay was performed according to previously described methods^54^.

### DNA methylation analysis

Genomic DNA was extracted from glioma cells and tissues and modified by bisulfite. Bisulfite conversion was performed by using an Epitect Bisulfite Kit (Qiagen). Bisulfite-treated DNA was amplified with BSP primers targeting the miR-708 promoter: 5′-GTTATTGAAGTTAATAACGGTGATTGATA-3′ (forward) and 5′-CTTCCTCGGAATTAACTATTACTGCG-3′ (reverse).

### ChIP assay

ChIP assay was performed using the EZ-CHIP Chromatin Immunoprecipitation Kit (Millipore, Billerica, MA, USA) according to the manufacturer’s protocol. The captured genomic DNA was collected and used for quantitative PCR analysis. The primers used to detect the miR-708 promoter sequence were listed in Supplementary Table S2.

### Statistical analysis

SPSS 13.0 and GraphPad Prism 5.0 software were used for statistical analysis. The values are shown as the mean ± SEM. Analyses of different groups were performed using one-way analysis of variance or two-tailed Student’s *t* test. The relationship between SPHK2 and miR-708 expression was quantified using Spearman’s correlation. *P* < 0.05 was considered statistically significant.

## Supplementary information

Supplementary data.
