# Supplementary material for: Silencing of microRNA-708 promotes cell growth and epithelial-to-mesenchymal transition by activating the SPHK2/AKT/β-catenin pathway in glioma
Source: Cell Death Dis. 2019 Jun 6;10(6):448. doi: 10.1038/s41419-019-1671-5 (PMC6554356; doi:10.1038/s41419-019-1671-5)
Supplement: Supplementary file 1 — Supplementary data. [file 41419_2019_1671_MOESM1_ESM.docx]

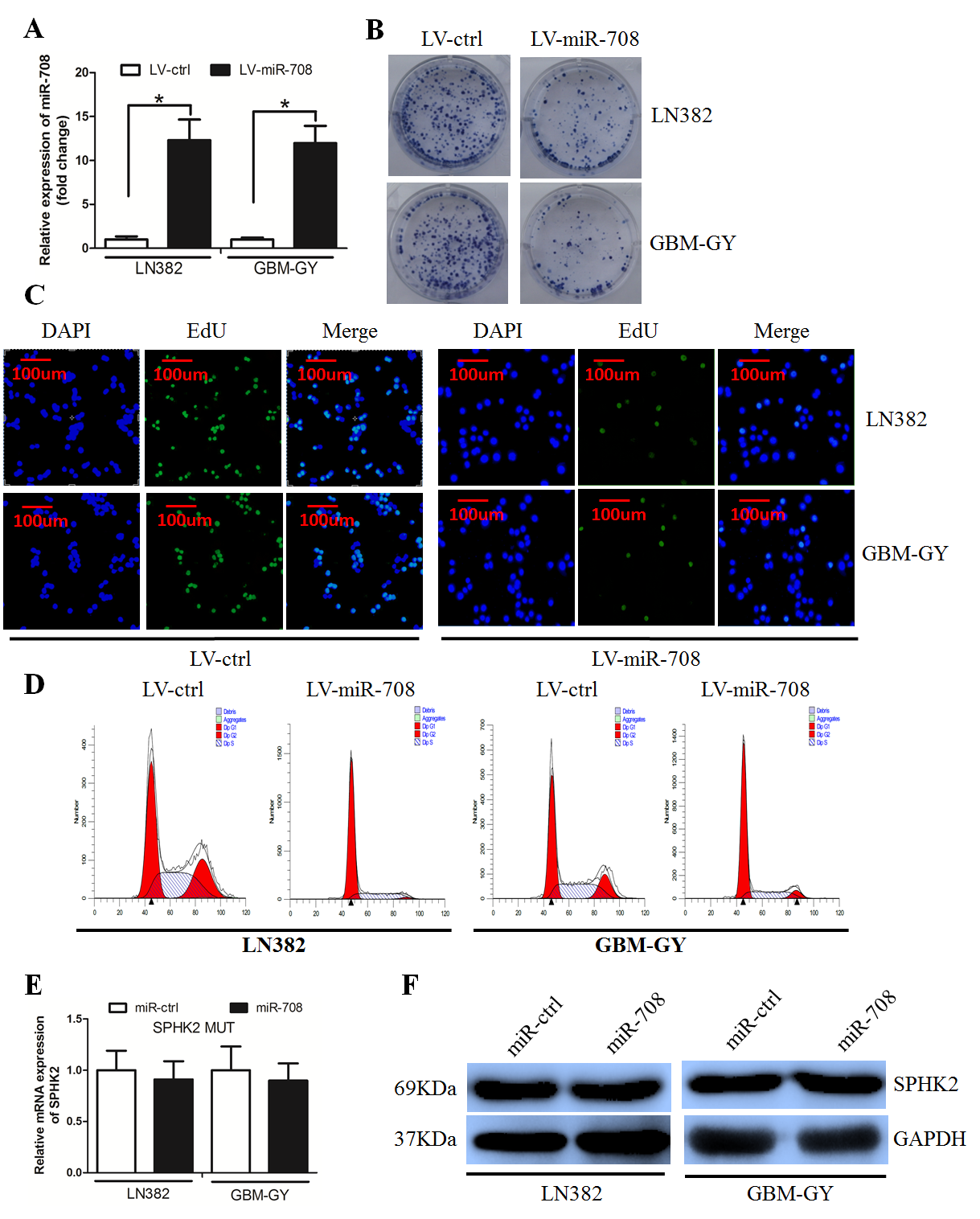


**Supplementary Figure 1 miR-708 decreased glioma cell growth in vitro**

(A) RT-PCR was used to validate the efficiency of miR-708 transduction. (B) Colony formation analysis showed that miR-708 overexpression inhibited cell proliferation. (C) The number of EdU-positive cells is lower in the LV-miR-708-treated group than that in the LV-ctrl-treated group. (D) miR-708 induced cell cycle arrest in the G1 phase. (E) and (F) When the biding sites of miR-708 on SPHK2 were mutant, the suppressive effects of miR-708 on SPHK2 mRNA and protein were abolished.


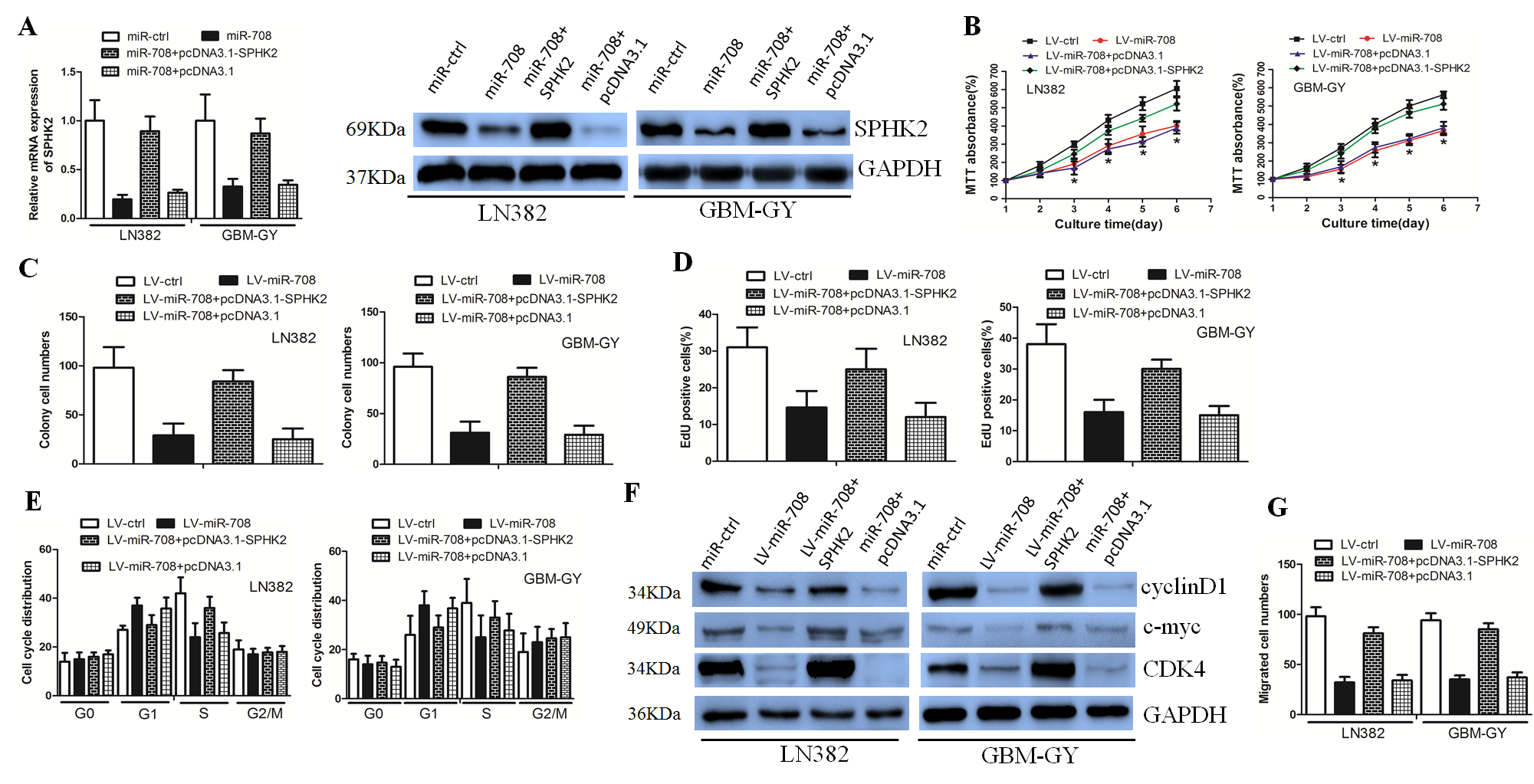


**Supplementary Figure 2 SPHK2 overexpression restored the tumor-suppressive effect of miR-708**

(A) Glioma cells treated with pcDNA3.1-SPHK2 vector show restored SPHK2 expression that had been decreased by miR-708. (B) MTT assays revealed that miR-708 inhibits cell growth, whereas SPHK2 overexpression resulted in the opposite effect. (C) Colony formation assays demonstrated that re-introduction of SPHK2 into LV-miR-708 cells inhibited the effects of miR-708. (D-F) The effects of miR-708 on cell proliferation and cell cycle distribution were restored by SPHK2 overexpression, as determined by EdU, flow cytometry, and western blot assays. (G) Boyden assay revealed that SPHK2 overexpression mitigated the effect of miR-708 on cell invasion.


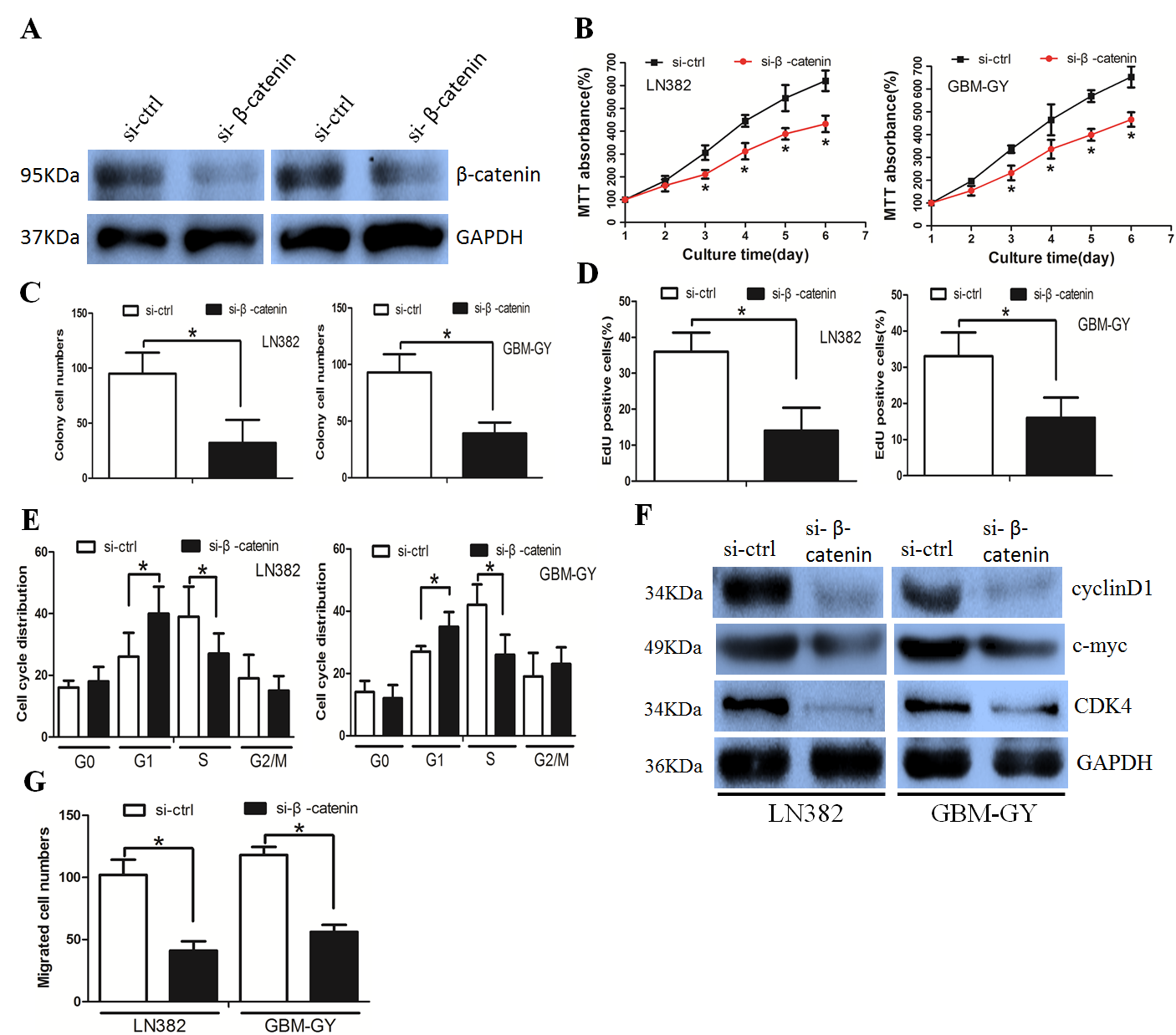


**Supplementary Figure 3β-catenin down-regulation mimics the effect of miR-708 on glioma cells**

(A)β-catenin was knocked down in glioma cell lines by siRNA. (B) β-catenin down-regulation inhibited LN382 and GBM-GY cell growth. (C)β-catenin inhibition impeded glioma cell colony formation ability. (D) The number of EdU-positive cells was lower in the si-β-catenin group when compared with the si-ctrl group. (E) β-catenin down-regulation contributed to cell cycle arrest in G1 phase. (F) β-catenin inhibition lead to the down-regulation of cyclin D1, c-myc, and CDK4. (G) **β** -catenin knockdown decreased glioma cell invasion.


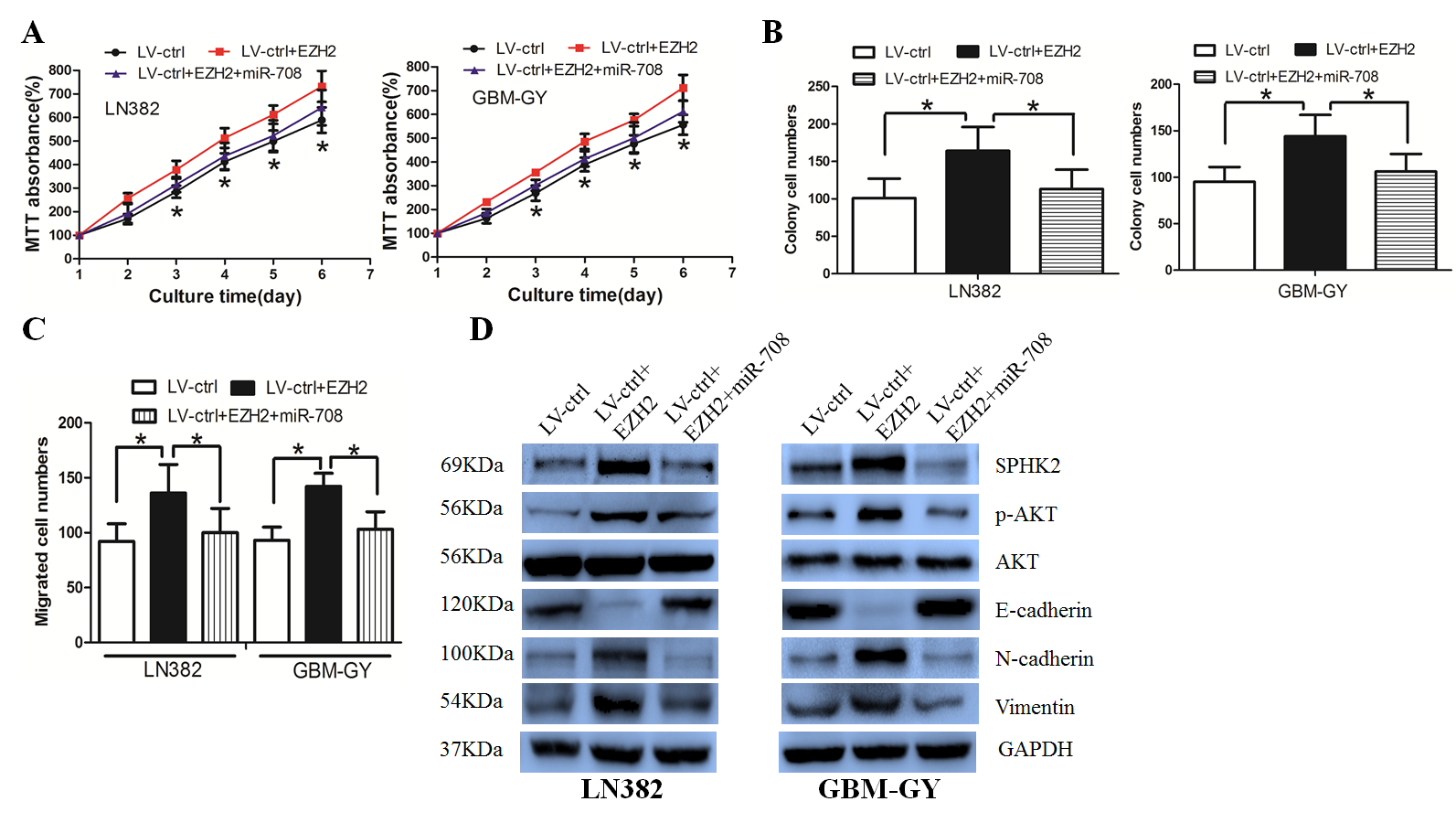


**Supplementary Figure 4**  **Restoration of miR-708 could counteract EZH2 overexpression effect on glioma cell growth and invasion**

(A) Overexpression of EZH2 accelerated the LN382 and GBM-GY cell lines growth, while overexpression of miR-708 counteracted this effect. (B) The colony formation ability was increased by EZH2, while was impeded by miR-708. (C) EZH2 increased glioma cell invasion ability, while miR-708 dismissed this effect. (D) EZH2 increased SPHK2 and p-AKT expression, and lead to EMT phenotype. Overexpression of miR-708 impeded EZH2’s effect.

**Supplementary Table 1 The relationship between clinicopathological characteristics and miR-708 expression in glioma patients.**

| Variables | NO. of cases | MiR-708 expression |  | P value |
| --- | --- | --- | --- | --- |
| Gender |  | High | Low |  |
| Male | 52 | 23 | 29 | 0.27 |
| Female | 47 | 26 | 21 |  |
| Age |  |  |  |  |
| ≤60 | 45 | 22 | 23 | 0.912 |
| ﹥60 | 54 | 27 | 27 |  |
| Histological type |  |  |  |  |
| OT | 15 | 9 | 6 | 0.183 |
| AT | 75 | 35 | 40 |  |
| Other | 9 | 5 | 4 |  |
| WHO grade |  |  |  |  |
| I-II |  | 23 | 12 | 0.017 |
| III-IV |  | 26 | 38 |  |

**Supplementary Table 2** Primers used in the study.

|  | Forward(5'-3') | Reverse(5'-3') |
| --- | --- | --- |
| miR-708 | GCTCGTCCCATCGGGGGTATTG | CCGCCGGTATTGCTTAAGCAAACG |
| SPHK2 | TATTCCTGGAGACGAGGGC | CTCAGACGTCAGAGGCTG |
| GAPDH | AACTTGTCAGTCCTGGACATG | AGTGCCTGTCGCTGTTTTAGT |
| Primers of miR-708 used in CHIP assay | | |
|  | Forward(5'-3') | Reverse(5'-3') |
| P1 | CTTGCAAATCCAGAGGCAGA | CGAGTCCCGGGCTCTTTA |
| P2 | GCCCACAGGTTTGCGGGAC | CGGGATAGCCCGACATTTGG |
| P3 | CGCTAAGTCATGCCCCTACC | GCCCACCATGGGTGGTTTAT |
| P4 | CCGGGCATAAACAGATTTAG | TTGGTCCCCGACAAAGG |
